# Empathy, Professional Quality of Life and Stress Coping Strategies Among Dental Hygienists—A Cross‐Sectional Study

**DOI:** 10.1111/idh.70070

**Published:** 2026-04-16

**Authors:** Nir Uziel, Efrat Gilon, Alina Haj, Ilana Eli

**Affiliations:** ^1^ Unit of Medical Education, Department of Oral Rehabilitation, Goldschleger School of Dental Medicine, Gray Faculty of Medicine & Health Sciences Tel Aviv University Tel Aviv Israel; ^2^ Goldschleger School of Dental Medicine, Gray Faculty of Medicine & Health Sciences Tel Aviv University Tel Aviv Israel

**Keywords:** burnout, compassion satisfaction, dental hygienists, empathy, ProQoL, secondary traumatic stress, stress coping strategies

## Abstract

**Introduction:**

Dental hygienists work in a stressful environment and often experience substantial personal distress, which can adversely affect their empathy as well as their professional quality of life. The present study attempted to evaluate stress coping strategies and their effect on empathy, burnout, compassion satisfaction and secondary traumatic stress among dental hygienists.

**Methods:**

A cross‐sectional online survey was conducted using the following self‐report questionnaires (i) Jefferson Scale of Physician Empathy for Health Professionals (JSPE‐HP); (ii) professional quality of life questionnaire (ProQoL) to measure burnout, compassion satisfaction and secondary traumatic stress; and (iii) Coping Inventory for Stressful Situations—Situation Specific Coping inventory (CISS‐SSC) to measure task‐focused coping, emotion‐focused coping and avoidance focused coping strategies.

**Results:**

127 hygienists responded. Subjects' empathy could be best predicted by task‐oriented coping style and burnout (stepwise linear regression, *R* ^2^ = 0.19, *p* < 0.01.). Subjects' burnout could be best predicted by emotion‐focused coping style, empathy and age (*R* ^2^ = 0.346, *p* < 0.001.), their level of secondary traumatic stress could be best predicted by emotional‐focused coping (*R* ^2^ = 0.283, *p* < 0.001) and level of compassion satisfaction could be best predicted by task‐focused coping style, emotion‐focused coping style and professional experience (*R* ^2^ = 0.315, *p* < 0.01).

**Conclusion:**

Findings suggest that in the dental hygiene profession, coping styles play a significant role in empathy and the different aspects of subjects' professional quality of life. While task‐coping style positively affects subjects' empathy, emotion‐focused coping causes dental hygienists to be more vulnerable to secondary traumatic stress and burnout.

## 1 Introduction

Empathy refers to the ability of a person to understand the feelings expressed by others and respond compassionately to another person's distress [1]. In health care, empathy refers to ideas of compassion, thoughtfulness, attentiveness and caring, which present a desirable type of ‘chairside manner’. Empathy can positively affect patients' adherence to medical regimens and increase satisfaction with treatment [2]. In the health professions, empathy is commonly evaluated through the Jefferson Scale of Physician Empathy—Health Professionals (JSPE) [3, 4].

Similar to dentists, dental hygienists' everyday work is intermingled with their patients' anxiety and distress. Occasionally, dental hygienists deal with severe phobic reactions to dental treatment that may originate from previous dental‐related trauma [5, 6]. Various terms have been used to describe the effect of others' suffering on their caregivers, such as vicarious traumatisation or secondary traumatic stress. Secondary traumatic stress addresses the vicarious influences of patients' pain and discomfort that influence caretakers. If not managed by appropriate strategies, this interpersonally‐derived strain may exhibit high stress levels, affecting the dental professional's physical and emotional health and eventually leading to burnout [7, 8].

An accepted measure to study aspects of professional work among caretaking professions is the Professional Quality of Life Scale (ProQoL) [9]. Professional quality of life incorporates two aspects: the positive (Compassion Satisfaction) and the negative (Compassion Fatigue). Compassion fatigue breaks into two parts. The first part concerns exhaustion, frustration, anger and depression typical of burnout. The second part is Secondary Traumatic Stress—a negative feeling driven by fear and work‐related trauma that can be primary or secondary [10]. The ProQoL evaluates the positive measures of work gratification and the negative aspects of professional life, like secondary traumatic stress and burnout, which is a well‐known problem in medical professions [9, 10]. Studies demonstrate high levels of burnout among physicians and nurses [11, 12], as well as dentists, dental staff, hygienists, dental and dental hygiene students [13, 14, 15, 16, 17, 18, 19, 20, 21, 22].

Coping is an important factor related to stress [23, 24, 25]. The Lazarus and Folkman [25] transactional model of stress and coping claims that coping is a process of an ongoing cognitive and behavioural effort to manage internal and external demands evaluated by the person as taxing or exceeding his/her resources. Three central coping strategies were proposed: task‐focused coping (TC), emotion‐focused coping (EC) and avoidance‐focused coping (AC) [25, 26]. TC concentrates on acting in order to change the circumstances that caused the distress (e.g., doing something to solve the problem); EC aims to regulate stress through an attempt to understand and express emotions and/or change the meaning of the event (e.g., self‐preoccupation) [27, 28], and AC is based on avoiding confrontations with the source of stress (e.g., distraction) [28, 29].

Reports showed that emotional coping may cause dentists to be vulnerable to burnout and secondary traumatic stress [22]. Regretfully, data concerning empathy, professional quality of life and stress coping strategies among the para dental personnel are relatively scarce. One‐third of the dental hygienists in California experience burnout [30]. Job satisfaction and burnout among dental hygienists are associated with feelings of frustration [31]. Compassion satisfaction appears to impact burnout and thoughts of leaving the profession among dental hygienists [32].

The present study aims to evaluate the relationships between professional quality of life, empathy and stress‐coping strategies among dental hygienists.

## 2 Materials and Methods

The study was based on convenience sampling. Invitations to participate in the survey were distributed in 2022 via posts in professional Facebook groups. To reduce selection bias, the questionnaire was posted in two Facebook groups of dental hygienists. Participation was voluntary and anonymous, and validated questionnaires were used to minimise measurement bias. Eligible to participate was any hygienist licensed to practice dentistry in Israel by the Ministry of Health. Hygienists who responded to the invitation opened a link to the questionnaire, including an informed consent form, and submitted their responses anonymously via a professional survey site (Alchemer.com).

The ethics committees of Tel Aviv University approved all the study procedures (No. 0002306‐2).

The following questionnaires were used:

1. *Professional profile and demographic data questionnaire*: The questionnaire collected demographic information as follows: gender (male/female); age (years); marital status (married/in significant relationship/single); country of birth; professional experience (no. of years in practice); workload (no. of working hours per week); workplace (private practice/public clinic/hospital/university, if more than one workplace—specify % of time in each one).
2. *The Jefferson Scale of Physician Empathy—Health Professionals (JSPE‐HP)* [3]:
  The JSPE‐HP is a 20‐item psychometrically validated scale to measure empathy and is used among physicians [33, 34] dental students [4], and recently dentists [35]. Participants rate their level of agreement on a 7‐point Likert scale, ranging from 1 (strongly disagree) to 7 (strongly agree). Results vary from a minimum of 20 to a maximum of 140 (the higher the score, the higher the participant's level of empathy) [3, 33].
  The internal consistency of the questionnaire in the present study (Cronbach *α*) was 0.80.
3. *Professional quality of life*: The Hebrew Version of the ProQoL Questionnaire Was Used [36].
  The questionnaire is well accepted and has been previously used in Israel [21, 22].
  The questionnaire includes 30 items, rated on a 1–5 Likert scale. It measures the following factors: [9, 10]
  - Professional burnout (BO).
  - Compassion satisfaction (CS), which refers to gratification generated by positive feelings about colleagues and the pleasure derived from helping others through work and from effectively completing work.
  - Secondary traumatic stress (STS), which refers to indirect work‐related exposure to stress and trauma due to interactions with patients.
  The internal consistency of the ProQoL subscales (Cronbach's *α*) was as follows: CS, 0.85; BO, 0.53; and STS, 0.62.
4. *Coping inventory*: Coping styles were measured by the short version of the Coping Inventory for Stressful Situations—Situation Specific Coping Inventory (CISS‐SSC) [23, 29], which is widely used [37, 38, 39]. The instrument includes 21 items, rated on a 5‐point Likert scale with seven items for each factor. Respondents are asked to rate the extent to which they employ each strategy when confronted with stress related to providing care to patients [9]. The Hebrew version used in the present study is based on a translation by Zevulun [40] and was previously used among dentists [22]; no significant discrepancies were found. The CISS‐SSC measures three coping dimensions:
  - Task‐focused coping (TC)—refers to direct actions to affect the situation and to reduce the stress it evokes (problem orientation).
  - Emotion‐focused coping (EC)—measures the efforts that are directed towards emotionally reframing the problem, so it elicits less stress (personal orientation) [41].
  - Avoidance‐focused coping (AC)—measures how much an individual tends to avoid confrontations with the source of stress.

The internal consistency of the CISS‐SSC subscales (Cronbach's *α*) was as follows: TC, 0.78; EC, 0.80; and AC, 0.79.

### 2.1 Statistical Analyses

Descriptive statistics (frequencies, means and standard deviations) were calculated for the variables. All quantitative variables were treated as continuous variables in correlation and regression analyses. No formal sensitivity analyses were conducted in this study. All analyses were based on the collected sample. Pearson's r correlations were used to analyse bivariate associations. Univariate analyses were performed to measure the underlying relationships between the independent variables (gender, age, years of experience, workload and coping styles) and the dependent variables (empathy, ProQoL subscale scores). The significance level was set at 0.05.

In the second stage, independent variables which showed significant correlation coefficients with empathy and/or ProQoL factors were subjected to linear regression analyses with empathy and each of the ProQoL factors as dependent variables.

The data were coded and analysed using SPSS version 23 (SPSS Inc., Chicago, IL, USA, 2015).

## 3 Results

The number of licensed hygienists in Israel, as recorded by the Israeli Ministry of Health in 2021, was 2769 [42]. It is, however, important to point out that there are no data regarding how many continue to work in the profession after graduation and obtaining an official licence. The survey was distributed through professional Facebook groups, one of which was a closed group for dental hygienists with 201 members, and another that included 1700 members.

One hundred seventy‐five subjects responded to the survey during the study period. Forty‐eight were excluded due to insufficient data supplied. Final analyses refer to 127 subjects (98.4% female). The study has sufficient power (> 80%) to detect moderate correlations (*r* = 0.25–0.30) between coping strategies and outcomes at *α* = 0.05.

Mean age of participants was 43.3 ± 11.1 years. 38.6% were born in Israel, with the rest born abroad (mainly in Russia or former USSR—39.4%). 83.5% were married or in a significant relationship. The average work experience was 15.5 ± 11.2 years, and the average workload was 28.8 ± 10.9 h per week. 34.6% were working in a single clinic, with the rest working in 2 or more places of work.

Data regarding the study variables are presented in Table 1.

**TABLE 1:** Empathy, professional quality of life and stress coping strategies (mean ± SD).

Variable	Mean	SD
Empathy (JSPE‐HP)^a^	115.96	12.60
Professional quality of life: (ProQoL)^b^ Burnout (BO)	24.10	4.60
Compassion satisfaction (CS)	40.90	6.00
Secondary traumatic stress (STS)	20.10	4.10
Stress coping strategies: (CISS‐SSC)^c^ Task coping (TC)	26.74	3.85
Emotional coping (EC)	16.52	4.83
Avoidance coping (AC)	18.97	5.25

^a^ Jefferson scale of physician empathy‐health professional.

^b^ Professional quality of life.

^c^ Coping inventory for stressful situations—situation specific coping.

### 3.1 Univariate Analyses

Subjects who were married or in a significant relationship experienced less secondary traumatic stress than single subjects (19.80 ± 4.00 vs. 21.90 ± 4.20 respectively, *p* < 0.05, *t*‐test). There were no differences in empathy, burnout or compassion satisfaction between married and single subjects. No differences in coping styles could be observed between married and single participants.

Subjects who worked in more than one place of work exhibited higher rates of secondary traumatic stress and a higher tendency for emotional coping than subjects working in only one clinic (STS, 20.9 ± 4.2 vs. 18.9 ± 3.7; EC, 17.36 ± 5.04 vs. 15.12 ± 4.13; *p* < 0.05 for both, *t*‐test). No differences between groups were observed for the other study variables.

Correlations among empathy, ProQoL sub‐scales and coping styles are presented in Table 2.

**TABLE 2:** Pearson correlation coefficients between the study variables.

	ProQoL^a^			CISS‐SSC^b^		
	BO	STS	CS	TC	EC	AC
BO (Burnout)						
STS (Secondary traumatic stress)	**0.474** **					
CS (Compassion satisfaction)	**−0.658** **	**−0.270** **				
TC (Task‐focused coping)	**−0.227** *	0.071	**0.389** **			
EC (Emotion‐focused coping)	**0.460** **	**0.532** **	**−0.385** **			
AC (Avoidance‐focused coping)	−0.05	0.044	**0.191** *			
Empathy^c^	**−0.325** **	−0.022	**0.285** **	**0.358** *	−0.079	−0.038
Workload (hours per week)	0.031	0.031	0.144	0.003	−0.160	−0.015
Age	**−0.214** *	−0.022	**0.211** *	0.095	−0.120	−0.135
Professional experience	**−0.248** ** *	−0.042	**0.280** **	0.137	**−0.221** *	−0.112

*Note:* Significant correlations marked in bold.

^a^ ProQoL—Professional quality of life.

^b^ CISS‐SSC—Coping inventory for stressful situations—situation specific coping.

^c^ No correlations were found between empathy and workload, age or years of professional experience.

* *p* < 0.05.

** *p* < 0.01.

### 3.2 Stepwise Linear Regression Analyses

#### 3.2.1 Empathy

Task‐oriented coping style and burnout significantly predicted subjects' empathy (Table 3). The overall regression was statistically significant (*R* ^2^ = 0.19, *F*(2,104) = 12.84, *p* < 0.01).

#### 3.2.2 Burnout

Emotion‐focused coping style, empathy and age significantly predicted subjects' burnout (Table 3). The overall regression was statistically significant (*R* ^2^ = 0.346, *F*(3,101) = 17.80, *p* < 0.001).

#### 3.2.3 Secondary traumatic stress

The best possible explanatory factor for STS was emotion‐focused coping (*R* ^2^ = 0.283, *F*(1,105) = 41.457, *p* < 0.001) (Table 3).

#### 3.2.4 Compassion satisfaction

Task‐focused coping style, emotion‐focused coping style and professional experience significantly predicted subjects' compassion satisfaction (Table 3). The overall regression was statistically significant (*R* ^2^ = 0.315, *F*(3,101) = 15.501, *p* < 0.01).

**TABLE 3:** Regression analyses for empathy, burnout, secondary traumatic stress and compassion satisfaction.

Model	Predictor	B	SE	Beta	*t*	*p*
Empathy	Task‐focused coping	0.348	0.106	0.296	3.287	0.001
	Burnout	−0.369	0.123	−0.271	−3.011	0.003
Burnout	Emotion‐focused coping	0.268	0.055	0.396	4.862	0.000
	Empathy	−0.227	0.058	−0.315	−3.902	0.000
	Age	−0.009	0.003	−0.207	−2.554	0.012
Secondary traumatic stress	Emotion‐focused coping	0.321	0.050	0.532	6.439	0.000
Compassion satisfaction	Task‐focused coping	0.393	0.092	0.355	4.268	0.000
	Emotion‐focused coping	−0.294	0.075	−0.331	−3.924	0.000
	Professional experience	0.010	0.005	0.183	2.141	0.035

## 4 Discussion

The American Institute of Stress defined occupational pressure as the leading source of stress for American adults and indicated that these have steadily increased over the past few decades [43]. Work‐related stress can lead to emotional exhaustion, depersonalisation and diminished personal accomplishment [44].

Previous studies investigated empathy, professional quality of life and coping strategies among Israeli dentists and dental assistants [21, 22, 45, 46]. The present study further expands the research to dental hygienists. Dentists' empathy (as measured by the JSPE‐HP questionnaire) was inversely associated with vicarious trauma [45] (a concept similar to secondary traumatic stress, measured by the Vicarious Trauma Scale [47]). Moreover, dentists' empathy correlated with the number of sleeping hours per night and increased with age [46]. Present results show that hygienists' empathy was not associated with workload, age or years of professional experience.

Although working in similar clinical settings and often encountering similar interpersonal problems, the two populations (dentists and dental hygienists) differ in several aspects. First, there are obvious academic and licensing differences, with dentists obliged to complete a full six‐year curriculum of academic studies and hygienists' licensing demanding merely a two‐year course of non‐academic studies. Additionally, the sample of Israeli dentists examined by Uziel et al. [45] included about 70% males, while the percentage of female dental hygienists in the present study (and in Israel) was close to 100%. Although no differences were found in the empathy scores of male and female dentists [45], the differences in gender distribution between the two studies might have contributed to the differences in empathy levels in the two populations.

Moreover, major differences exist in Israel in the mode of employment and work between dentists and dental hygienists. Dentists are independent and are responsible for their clinical decisions and treatment performances. Dental hygienists have to work under the direct (or indirect) supervision of a dentist who is in charge of the clinic (private or public). All these differences might have caused that while the best predictor of dentists' empathy was number of sleeping hours per night, vicarious trauma and age, the best predictors of empathy among hygienists were task‐oriented coping style and burnout.

Burnout can be detrimental to any occupation, but its effect on health caretakers should be a special concern. In California, approximately one‐third of the dental hygienists experienced burnout [30]. A major predictor of burnout among Californian hygienists was a lack of appreciation from management. Hygienists who felt appreciated at work experienced less emotional exhaustion and less depersonalisation. Additionally, compassion satisfaction impacted burnout and thoughts of leaving the dental hygiene profession [32].

In the present study, hygienists' burnout was best predicted by emotion‐focused coping, empathy and age. Emotion‐focused coping also played a significant role in the prediction of hygienists' compassion satisfaction and secondary traumatic stress. These findings resemble the findings among the dentists who work in close collaboration with this population [22].

Present results are in line with a recent work by Shoji et al. [48] who studied Japanese nurses and doctors and showed an association between empathy and lower burnout and between empathy and higher compassion satisfaction. Among the Japanese population, coping strategies were associated with higher compassion satisfaction and lower burnout [48].

The negative association between burnout and empathy, found in the present study, is similar to the findings among the medical staff in various countries [49]. Several studies showed that coping can mediate the relationship between burnout and secondary traumatic stress in caregivers in other parts of the world [50, 51]. Personal orientation directed towards emotionally reframing an encountered problem significantly affects dental professionals' professional quality of life, whether dentists or dental hygienists. In a study on emergency department healthcare professionals, emotion‐focused coping significantly predicted burnout characteristics (higher emotional exhaustion, higher depersonalisation and lower personal accomplishment, using the MBI questionnaire). In comparison, task‐focused coping significantly predicted lower emotional exhaustion, lower depersonalisation and higher personal accomplishment. Task‐oriented coping was associated with higher personal accomplishment and lower levels of burnout [52].

Chapman et al. [53] characterised the situations associated with negative emotions in the dental practice as belonging to either anxiety/fear, anger, disgust/guilt/shame or sadness/depression groups of emotions. Anxiety, which is associated with a sense of not being in control in clinical situations, anxious patients, striving to maintain high standards and managing workload might be experienced more frequently by dentists than by hygienists, whose span of treatments is more limited. The same can also be correct regarding the feeling of anger (provoked mainly by a sense of injustice about patient complaints and failure to achieve self‐imposed standards of perfection). Both groups (dentists and hygienists) might be aggravated by patient behaviours (lack of cooperation, failing to keep appointments, etc.). Apparently, for both groups, the cumulative effect of such feelings is mainly affected by their coping strategies in general and emotion‐focused strategy in particular.

### 4.1 Study Limitations

Using an internet sample and a relatively low response rate may have resulted in a biased sample not necessarily representative of the population of dental hygienists in Israel. The almost total female dominance among dental hygienists in Israel may not be comparable to that in other countries with more male dental hygienists. Thus, applying the results to other countries should be carried out with extra care. Additional studies are required to examine this issue. In‐depth interviews with dental hygienists and the dentists they work with can shed more light on this concern.

### 4.2 Conclusions

Findings indicate that coping styles significantly influence empathy and various dimensions of professional quality of life among individuals in the dental hygiene field. While task‐coping style positively affects subjects' empathy, emotion‐focused coping causes dental hygienists to be more vulnerable to secondary traumatic stress and burnout. Additionally, coping styles play significant role in subjects' positive feelings of gratification from work (compassion satisfaction).

## 5 Clinical Relevance

*Scientific rationale*: High‐pressure work environment can compromise dental hygienists' professional performance as well as personal well‐being.

*Principal findings*: Coping styles significantly affect dental hygienists' empathy and professional quality of life.

*Practical implications*: Better understanding of the coping mechanisms that help one to deal with stress and uncomfortable emotions can help dental hygienists to cope with professional stress more effectively. Self‐awareness, support and intentional behaviour are key to changing coping styles. Consequently, an increased emphasis on students' coping styles should be integrated into the educational curriculum for dental hygienists, as well as throughout their professional journey.

## Author Contributions

All authors gave their final approval and agreed to be held accountable for all aspects of the work. N.U. and E.G. conceived the ideas, supervised the study and wrote original Draft. A.H. collected the data and wrote original draft. N.U. and I.E. analysed the data and critically revised the manuscript. I.E. led the writing and editing.

## Funding

The authors have nothing to report.

## Conflicts of Interest

The authors declare no conflicts of interest.

## Data Availability

The data that support the findings of this study are available from the corresponding author upon reasonable request.
